# CHALLENGES FACING PRIMARY CARE NURSE PRACTITIONERS CARING FOR PERSONS LIVING WITH DEMENTIA

**DOI:** 10.1093/geroni/igae098.3826

**Published:** 2024-12-31

**Authors:** Kyle Featherston, Maura Dougherty, Siqing Wang, Lusine Poghosyan

**Affiliations:** Columbia University School of Nursing, New York, New York, United States; Columbia University School of Nursing, New York, New York, United States; Columbia University School of Nursing, New York, New York, United States; Columbia University School of Nursing, New York, New York, United States

## Abstract

The nurse practitioner (NP) workforce plays a vital role in caring for the growing population of persons living with dementia (PLWD). However, organizational and structural barriers hinder their contribution to patient care. This study presents results from a national sample of 968 primary care NPs who care for PLWD. NPs were asked to report on the organizational support and resources available in their practices to care for PLWD. NPs also reported on the relationships between NPs and administration, as well as NP job satisfaction, burnout, and intentions of leaving their jobs. NPs reported their organizations lacked the resources to care for PLWD that they had for patients with other chronic diseases. Specifically, only 14.67% reported a registry of PLWD overdue for services and fewer than 60% had tools, templates, and reminders for PLWD that were largely available for chronic conditions like hypertension and dementia. Crucially, these deficits in the organizational context of care resulted in NPs rating the quality of the care provided to PLWD at their organization lower than the overall quality of care the organization delivered. Although NPs were largely satisfied with their jobs, over one-third reported being burned out, and over one-fifth reported they intended to leave their jobs within the next year. Addressing these barriers to care and creating work environments conducive to NP practice are necessary to fully utilize the capacity of the NP workforce to improve care of the older adult population living with dementia.

